# Minimal contribution of ERK1/2-MAPK signalling towards the maintenance of oncogenic GNAQ^Q209P^-driven uveal melanomas in zebrafish

**DOI:** 10.18632/oncotarget.9207

**Published:** 2016-05-06

**Authors:** Mai Abdel Mouti, Christopher Dee, Sarah E. Coupland, Adam F.L. Hurlstone

**Affiliations:** ^1^ Faculty of Life Sciences, The University of Manchester, Manchester, United Kingdom; ^2^ Department of Molecular and Clinical Cancer Medicine, Institute of Translational Medicine, University of Liverpool, Liverpool, United Kingdom

**Keywords:** GNAQ, uveal melanoma, ERK1/2, p53, zebrafish

## Abstract

Mutations affecting Gαq proteins are pervasive in uveal melanoma (UM), suggesting they ‘drive’ UM pathogenesis. The ERK1/2-MAPK pathway is critical for cutaneous melanoma development and consequently an important therapeutic target. Defining the contribution of ERK1/2-MAPK signalling to UM development has been hampered by the lack of an informative animal model that spontaneously develops UM. Towards this end, we engineered transgenic zebrafish to express oncogenic GNAQ^Q209P^ in the melanocyte lineage. This resulted in hyperplasia of uveal melanocytes, but with no evidence of malignant progression, nor perturbation of skin melanocytes. Combining expression of oncogenic GNAQ^Q209P^ with p53 inactivation resulted in earlier onset and even more extensive hyperplasia of uveal melanocytes that progressed to UM. Immunohistochemistry revealed only weak immunoreactivity to phosphorylated (p)ERK1/2 in established uveal tumours—in contrast to strong immunoreactivity in oncogenic RAS-driven skin lesions—but ubiquitous positive staining for nuclear Yes-associated protein (YAP). Moreover, no changes were observed in pERK1/2 levels upon transient knockdown of GNAQ or phospholipase C-beta (PLC-β) inhibition in the majority of human UM cell lines we tested harbouring GNAQ mutations. In summary, our findings demonstrate a weak correlation between oncogenic GNAQ^Q209P^ mutation and sustained ERK1/2-MAPK activation, implying that ERK1/2 signalling is unlikely to be instrumental in the maintenance of GNAQ^Q209P^-driven UMs.

## INTRODUCTION

Uveal melanoma (UM), the most prevalent primary malignancy of the human eye, originates principally from melanocytes residing within the choroid. While effective treatments are available for primary tumours, approximately half of patients will develop metastases within 15 years following diagnosis, for which currently there is no effective treatment [1, 2]. This has created a pressing need to understand the molecular pathogenesis of the disease. Earlier studies had focused on the contribution of ERK1/2-MAPK pathway to UM development, since this pathway was implicated in cutaneous melanoma (CM) [3] that has some obvious parallels to UM. However, mutations in well-known CM oncogenes such as BRAF or NRAS that are responsible for constitutive ERK activation are conspicuously rare in UM [4, 5]. Major advances in our understanding of the molecular events that drive UM initiation and progression have subsequently arisen through re-sequencing UM genomes, resulting in the identification of several candidate oncogenes and tumour suppressor genes. Recurrent hypermorphic mutations of GNAQ or GNA11 (GNAQ/11), highly related Gαq proteins, mainly affecting Glutamine 209 have been detected in UM and are proposed as indirect activators of ERK signalling via phospholipase C-beta (PLC-β)-mediated activation of protein kinase C (PKC) [6–8]. However, GNAQ mutation and ERK activation are not correlated in clinical specimens [9], and clinical trials have so far shown little or no activity for PKC or MEK antagonists in patients with metastatic UM, prompting the question as to whether there may be other more relevant targets. Recent studies indicate that activation of the transcription factor Yes-associated protein (YAP), independently of PLC-β, appears to sustain proliferation and survival in UM cells [10, 11].

GNAQ/11 mutations do not correlate with clinical, pathological, and genetic indicators of tumour progression [7, 12], suggesting that they are early events in UM development and insufficient for malignant progression. Consistent with this notion, forced expression of GNAQ^Q209L^ was insufficient to transform primary melanocytes [6]. In keeping with all other solid cancers, multiple genetic and epigenetic changes likely underlie progression of uveal melanocytic neoplasia from benign hyperproliferation to infiltrative and metastatic spread. In UM, loss-of-function BRCA1 associated protein-1 (BAP1) mutations correlate with metastatic behaviour [13]. A significant body of evidence also indicates that the p53 pathway is abrogated in UM, and that the principal mechanism is likely the up-regulation of MDM2. Strong immunostaining of nuclear MDM2 was detected in 31/32 (97%) of primary UM, but not normal uveal melanocytes [14]. In further support, transcriptome profiling of a human UM cell line, followed by validation on a larger set of UM cell lines, implied the abrogation of a p53-driven gene expression program in UM cells, as compared to normal choroidal melanocytes [15]. Finally, functional inactivation of signalling downstream of p53 was detected in UM cells following irradiation, possibly explaining the radioresistance of UM [16].

UM is a rare cancer, and hence clinical trials to determine the efficacy of novel drugs are difficult to organize, requiring multicentre collaboration. Animal models are thus an invaluable patient surrogate, which can be used not only to investigate factors involved in pathogenesis, but also to examine responses to therapy. A number of transplantation models are available that model UM including patient-derived xenografts (PDX) [17]; however, transplantation models suffer from altered tumour cell behaviour due to *in vitro* adaptation, as well as a requirement for immune suppression in the host. The limitations of transplantation models can be addressed in genetically engineered animal models spontaneously developing UM. Previously, we and others have succeeded in engineering zebrafish model of CM through targeting expression of oncogenic RAS and BRAF to the melanocyte lineage [18, 19]. These models have generated considerable insights into the pathogenesis of CM [20–22], and also yielded a novel clinical drug candidate [23]. Herein, we describe the generation of transgenic zebrafish expressing oncogenic GNAQ^Q209P^ in the melanocyte lineage, yielding a model of benign uveal melanocytic hyperplasia which confirms the selective role of Gαq proteins in driving the proliferation of uveal melanocytes. To test whether oncogenic Gαq signalling co-operates with abrogation of p53 function in promoting disease progression, GNAQ^Q209P^ was misexpressed in a p53 loss-of-function genetic background. This resulted in the generation of an experimental model of UM progression that likely reflects the contribution of MDM2 overexpression in human UM. Moreover, we present data that calls into question the contribution of oncogenic GNAQ to ERK activation in UM.

## RESULTS

### Construction of a Tol2-based transposon oncogenic GNAQ^Q209P^ transgene

As a first step toward creating a genetically engineered zebrafish model of UM, human and zebrafish GNAQ protein sequences were aligned using BLAST software. This revealed a highly conserved GNAQ orthologue in zebrafish (Supplementary Figure S1). In order to drive oncogenic GNAQ^Q209P^ expression in zebrafish choroidal melanocytes from a transgene, it was first necessary to identify an appropriate promoter. We previously used a fragment from the *mitfa* promoter to direct oncogene expression to zebrafish cutaneous melanocytes, which resulted in CM development [18]. In embryos, endogenous *mitfa* is expressed not only in cutaneous melanocytes, but also in uveal melanocytes and the retinal pigmented epithelium (RPE) [24, 25]. However, the *mitfa* promoter fragment, employed by us and others, drives detectable reporter expression only in melanocytes [26]. Moreover, we observed that *mitfa* nullizygous (*nacre*) zebrafish lack choroidal melanocytes, but as previously reported [24], the RPE appears structurally normal (Supplementary Figure S2).

**Figure 1:**
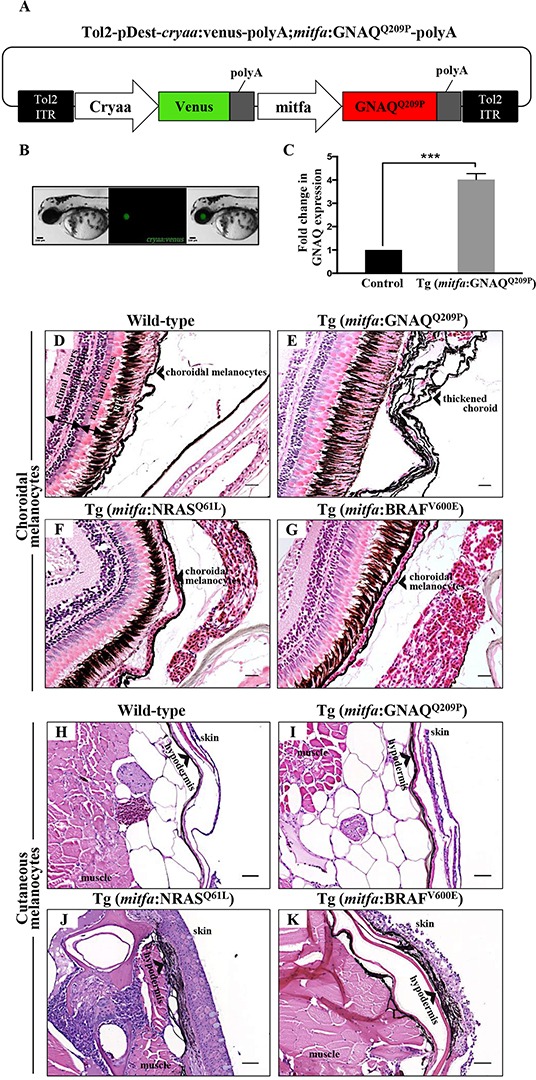
Only oncogenic GNAQ^Q209P^ is sufficient to induce choroidal melanocyte hyperplasia **A.** Schematic representation of elements in the Tol2-based transposon vector driving the expression of oncogenic GNAQ^Q209P^ under the control of zebrafish *mitfa* promoter in the melanocyte lineage and Venus fluorescent reporter under the control of *cryaa* promoter in the eye lens. Abbreviations: ITR, inverted terminal repeat. **B.** Example of a 5 dpf transgenic zebrafish embryo with a fluorescent eye lens. Scale bar, 100 μm. **C.** RT-qPCR data showing a 4.1 fold increase in GNAQ expression in the melanocytes of 2-month-old F_1_ Tg (*mitfa*:GNAQ^Q209P^) zebrafish, as compared to non-injected controls. Data represents mean ± SEM of triplicates of three independent experiments. *** P <0.05 using two-tailed, unpaired t test. **D, E, F, G.** H&E staining of transverse sections of formalin-fixed and paraffin-embedded eye specimens of control wild-type, Tg (*mitfa*:GNAQ^Q209P^), Tg (*mitfa*:NRAS^Q61L^), and Tg (*mitfa*:BRAF^V600E^) zebrafish, respectively. Choroidal hyperplasia observed in the thickened choroid (E; black arrowhead) was only observed in transgenic animals expressing oncogenic GNAQ^Q209P^. In contrast, as compared to control wild-type **H.** and Tg (*mitfa*:GNAQ^Q209P^) **I.** hyperplasia of cutaneous melanocytes (black arrowhead) was only detected in transverse sections of the torso region of Tg (*mitfa*:NRAS^Q61L^) **J.** and Tg (*mitfa*:BRAF^V600E^) **K.** zebrafish. Abbreviations: RPE, retinal pigmented epithelium. Scale bars, 20 μm.

To generate oncogenic GNAQ cDNA, wild-type zebrafish GNAQ coding sequence was mutagenized to substitute the conserved Glutamine (Q) at position 209 with Proline (P). Glutamine 209 of GNAQ is similar to Glutamine 61 of RAS proteins: substitution results in a conformational change, leading to the loss of intrinsic GTPase activity and sustained signalling. We then constructed a Tol2-based transposon vector incorporating the *mitfa* promoter fragment and oncogenic GNAQ^Q209P^ cDNA (Figure 1A). The transposon construct also comprised a second expression cassette that allowed the rapid identification of transgene carriers through driving Venus GFP expression in the eye lens (Figure 1B). Following sequence verification, the GNAQ^Q209P^ construct was co-injected with Tol2 transposase mRNA into one-cell stage wild-type zebrafish embryos. Potential founders were identified while embryos by screening for GFP expression in the lens, and then grown and mated to wild-type zebrafish to generate a first filial F_1_ generation (again identified by GFP positive lenses). To validate the expression of the oncogene, RNA was extracted from dissected caudal fins of 2-month-old F_1_ adult transgenic zebrafish then converted to cDNA, which was used as a template for real-time quantitative reverse transcription PCR (qRT-PCR) analysis. This revealed a 4.1 fold increase in GNAQ expression in transgenic zebrafish, as compared to non-injected controls (Figure 1C).

### *Mitfa*-driven expression of oncogenic GNAQ^Q209P^ induces hyperproliferation of choroidal melanocytes

Cohorts of wild-type and transgenic F_1_ adult fish were sacrificed at two and five months of age for histological examination of the choroid. No changes were apparent at two months (data not shown), but multi-layering of the choroid was detected in approximately 65% (15/23) of five-month-old Tg (*mitfa*:GNAQ^Q209P^) zebrafish, with no structural or numerical aberrations detected in the RPE (Figure 1E); also no evidence of infiltration or perforation of the sclera was observed. We posited that the choroidal hyperplasia observed in F_1_ animals involving the entire uveal tract might be likened to ocular melanosis/melanocytosis in man (although this is not associated with GNAQ mutation [27]). The pathology was completely benign, as malignancy was not observed in these animals even after a year (data not shown).

While CMs frequently harbour activating mutations in either NRAS or BRAF genes, the vast majority of UMs arise independently of these two oncogenic mutations [28]. On the contrary, GNAQ mutations occur at a high frequency in UM, but are rarely detected in their cutaneous counterparts. Using transgenic zebrafish lines expressing oncogenic BRAF (BRAF^V600E^), NRAS (NRAS^Q61L^), or GNAQ (GNAQ^Q209P^) in the melanocyte lineage under the control of *mitfa* promoter, we attempted to directly contrast the ability of oncogenic GNAQ^Q209P^ on the one hand and NRAS^Q61L^ and BRAF^V600E^ on the other to induce proliferative responses in uveal versus cutaneous melanocytes. Adult wild-type and transgenic zebrafish were euthanized and their histology was examined. In contrast to the choroidal hyperplasia observed in Tg (*mitfa*:GNAQ^Q209P^) zebrafish, choroidal melanocytes were morphologically normal in all specimens of Tg (*mitfa*:NRAS^Q61L^) (Figure 1F) and Tg (*mitfa*:BRAF^V600E^) (Figure 1G) zebrafish examined (for each, n>20). In contrast, hyperplasia of cutaneous melanocytes was evident in the hypodermis of Tg (*mitfa*:NRAS^Q61L^) (Figure 1J) and Tg (*mitfa*:BRAF^V600E^) (Figure 1K) zebrafish, but not in Tg (*mitfa*:GNAQ^Q209P^) zebrafish (Figure 1I). Thus, we conclude that zebrafish uveal melanocytes and cutaneous melanocytes appear to respond distinctly to different recurrently-mutated melanoma oncogenes, mirroring the pattern of genetic lesions detected in human UM and CM.

### GNAQ^Q209P^-driven hyperplastic lesions demonstrate restricted activation of ERK and YAP signalling

**Figure 2:**
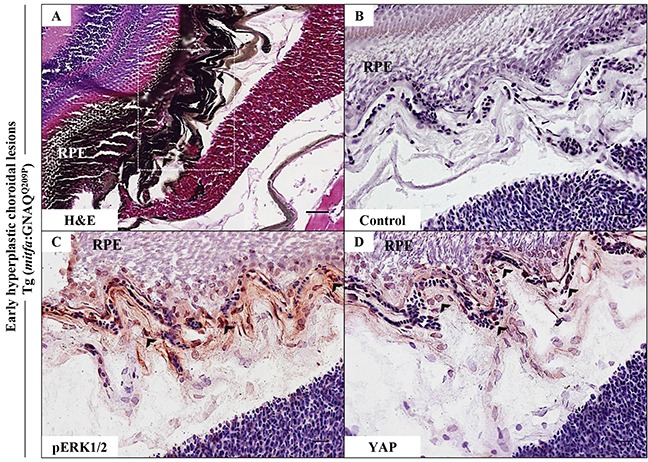
Oncogenic GNAQ^Q209P^-mediated activation of ERK and YAP signalling in choroidal melanocytes at the junction between RPE and choroid **A-D.** Transverse sections of formalin-fixed and paraffin-embedded eye tissues of F_1_ generation, 5-month-old Tg (*mitfa*:GNAQ^Q209P^) zebrafish. (A) H&E staining demonstrating choroidal hyperplasia (black arrowheads). White dashed box indicates the region of the choroid magnified in B-D. (B-D) Transverse sections of formalin-fixed and paraffin-embedded eye tissues were stained by IHC, visualized by ImmPact NovaRed peroxidase (HRP) substrate then counterstained with hematoxylin (blue). (B) Negative control: section incubated with 1x PBS instead of primary antibody. (C) Immunoreactivity to pERK1/2 (read-out of ERK activation; black arrowheads) in melanocytes at the interface between the RPE and choroid. (D) YAP-positive nuclei (read-out of YAP activation; black arrowheads) in the same cells. Scale bars, 20 μm.

Activation of ERK1/2-MAPK signalling by oncogenic GNAQ mutations was first demonstrated in *vitro* by transient expression of mutant GNAQ^Q209L^ construct in hTERT/CDK4R24C/p53DD melanocytes, which resulted in increased expression of phosphorylated ERK (pERK), as compared to the same melanocytes transfected with GNAQ^WT^ or empty vector [6]. In addition, recent findings demonstrated the activation of transcription factor YAP in UM by GNAQ/11 oncogenes and highlighted its fundamental role in the maintenance of uveal tumours [10, 11]. Thus, to address the functional conservation of oncogenic GNAQ expression in our zebrafish model, hyperplastic lesions were examined for ERK and YAP activation by immunohistochemistry (IHC). For examining ERK status, hyperplastic lesions were stained with anti-pERK1/2 polyclonal antibody that detects activated ERK1 and ERK2 when phosphorylated at residues corresponding to Thr202/Tyr204 and Thr185/Tyr187 in man respectively. IHC revealed positive immunoreactivity to pERK1/2 only within melanocytes at the interface between the RPE and choroid, where capillaries are concentrated (Figure 2C). Although less marked, YAP-positive nuclei were apparent at the same junctional zone (Figure 2D) — nuclear localization is critical for YAP-mediated oncogenic effects. Thus, the above findings indicate that zebrafish oncogenic GNAQ^Q209P^ can activate ERK1/2-MAPK and YAP signalling pathways, albeit restricted to melanocytes occupying a niche at the junction between the RPE and choroid. This indicates conserved functions of oncogenic GNAQ in zebrafish and man, and validates the transgenic line as a model of UM induction by oncogenic GNAQ.

### GNAQ^Q209P^ expression restores pigmentation in melanocytes and RPE of *golden* zebrafish, and again with evidence of choroidal hyperplasia

**Figure 3:**
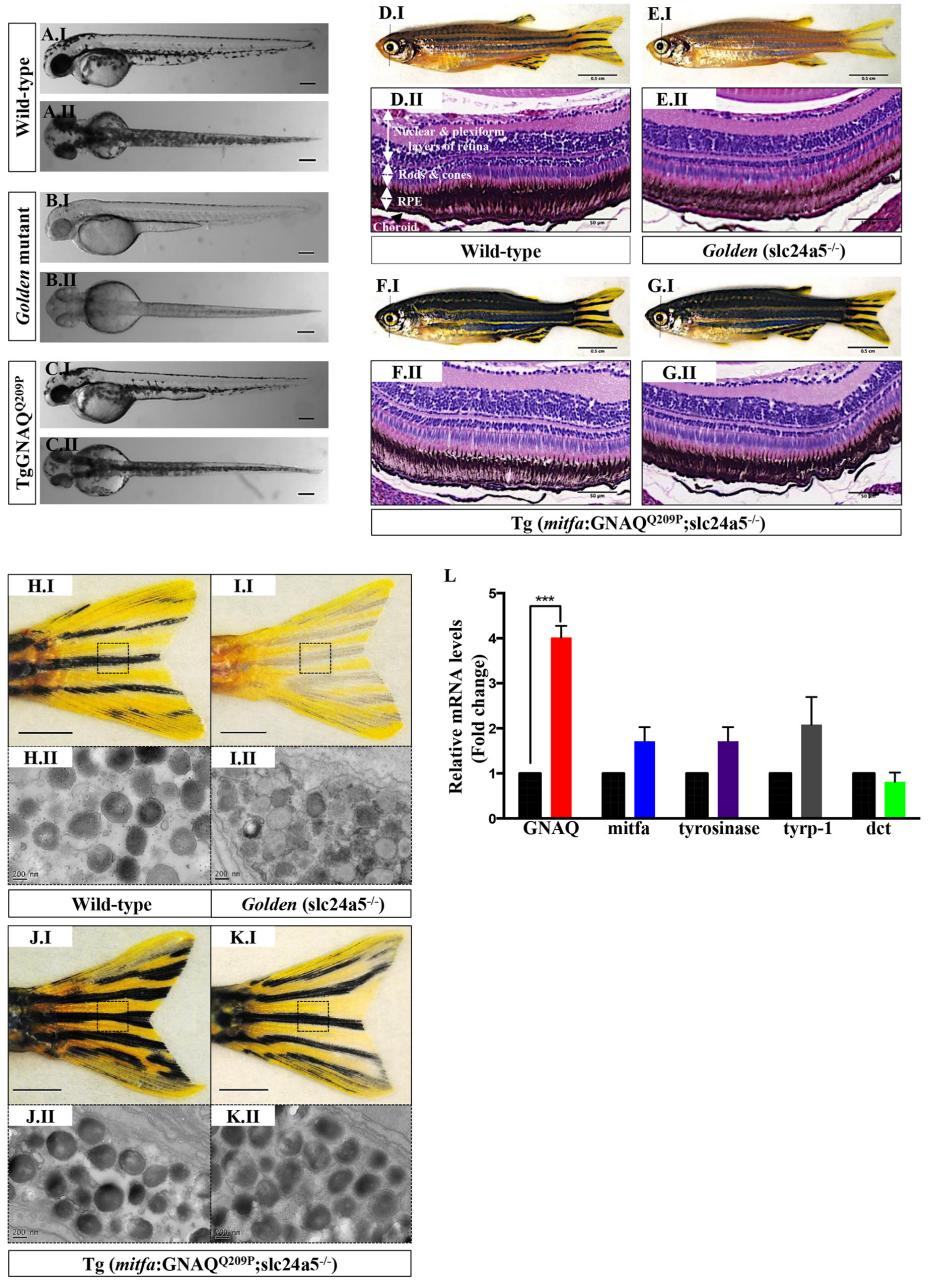
GNAQ^Q209P^ rescues the hypopigmentation phenotype of *golden* (slc24a5^−/−^) zebrafish **A.I, B.I, C.I.** Lateral views of wild-type, *golden*, and Tg (*mitfa*:GNAQ^Q209P^;slc24a5^−/−^) 5 dpf zebrafish embryos, respectively. **A.II, B.II, C.II.** Top views of wild-type, *golden*, and Tg (*mitfa*:GNAQ^Q209P^;slc24a5^−/−^) 5 dpf zebrafish embryos, respectively. Scale bars, 100 μm. **D.I, E.I, F.I, G.I.** Lateral views of 2-month-old adult zebrafish. (D.I) Wild-type illustrating normal melanin pigmentation in skin melanocytes. (E.I) Light-skinned *golden* mutant (slc24a5^−/−^) with stripes comprising less melanin-rich melanocytes. (F.I, G.I) Two examples of Tg (*mitfa*:GNAQ^Q209P^;slc24a5^−/−^) zebrafish from independent founders, acquiring darker stripes as compared to wild-type (D.I) and *golden* mutants (E.I), but still conserving the normal striped pigment pattern. Scale bars, 0.5 cm. **D.II, E.II, F.II, G.II.** H&E staining of transverse sections of formalin-fixed and paraffin-embedded eye specimens of 2-month-old adult zebrafish. (D.II) Normally pigmented RPE located between the photoreceptors layer of the retina (rods & cones) and the choroid in a wild-type zebrafish. (E.II) Lightly pigmented RPE in a *golden* mutant. (F.II, G.II) Rescuing of hypopigmented RPE in both GNAQ^Q209P^-expressing *golden* mutants, with no pathological findings yet observed in the choroidal melanocyte layer. Scale bars, 50 μm. **H.I, I.I, J.I, K.I.** Tail fins of wild-type, *golden,* and two Tg (*mitfa*:GNAQ^Q209P^;slc24a5^−/−^) zebrafish, respectively. These were dissected, fixed, and sectioned for TEM analysis. Black dashed boxes indicate representative regions of the tail fins examined by TEM. Scale bars, 0.5 cm. **H.II, I.II, J.II, K.II.** Transmission electron micrographs illustrating the structure of melanosomes in the melanocytes of dissected tail fins. Melanosomes in *golden* mutants (I.II) are less densely pigmented as compared to those of wild-type (H.II) and transgenic zebrafish (J.II, K.II). Scale bars, 200 nm. **L.** Relative to zebrafish EF1alpha (EF1α) expression and as compared to non-injected *golden* controls, qRT-PCR data show significant increase in mRNA expression levels of GNAQ in Tg (*mitfa*:GNAQ^Q209P^;slc24a5^−/−^) zebrafish. In contrast, no significant alterations were observed in the expression of mitfa or downstream differentiation genes including tyrosinase, tyrp-1, and dct. Results represent mean ± SEM of triplicates of three independent experiments. ***P <0.05, using two-tailed, unpaired t test. Abbreviations: tyrp-1, tyrosinase-related protein 1; dct, dopachrome tautomerase.

Concerned that IHC on any resultant pigmented neoplastic lesions might be compromised by the presence of melanin and the necessity to bleach sections, we injected the GNAQ^Q209P^ transgene construct into zebrafish zygotes of *golden* mutants characterized by delayed and reduced melanin pigmentation in skin melanocytes and RPE, as a result of a loss-of-function mutation in the slc24a5 gene [29]. To our initial surprise, pigmentation was restored in transgenic embryos (that is those with fluorescent green lenses) starting at day 2 post-fertilization (Figure 3C.I, 3C.II), and persisted in 49% of adult F_0_ transgenic zebrafish. Subsequent backcrossing from F_0_ founders to *golden* mutants resulted in an F_1_ generation with stable transgene incorporation, in which pigmentation was uniformly rescued (Figure 3F.I, 3G.I), and were visibly darker than non-injected *golden* (Figure 3E.I) and even wild-type (Figure 3D.I) zebrafish. Moreover, histological examination of transverse sections of eye tissues of 2-month-old GNAQ^Q209P^-expressing zebrafish revealed pigmentation-rescued RPE (Figure 3F.II, 3G.II), as compared to non-injected *golden* mutants (Figure 3E.II). Transmission electron microscopy (TEM) confirmed the presence of abundant melanin-rich melanosomes in the melanocytes of dissected tail fins of GNAQ^Q209P^–expressing transgenics (Figure 3J.II, 3K.II), which as previously documented [29], were less densely pigmented in *golden* mutants (Figure I.II). qRT-PCR, which again confirmed increased expression of GNAQ in transgenic zebrafish, failed to reveal significant changes in the mRNA levels of key pigmentation genes such as mitfa, tyrosinase, tyrosinase-related protein-1 (tyrp1), or dopachrome tautomerase (dct), which might otherwise have accounted for the increased melanin synthesis (Figure 3L). Thus, we concluded that forced expression of GNAQ^Q209P^ in zebrafish melanocytes stimulates melanin production, although the mechanism is still unclear.

Notwithstanding the hyperpigmentation of GNAQ^Q209P^-expressing zebrafish, which clearly demonstrated the activity of the transgene product throughout the melanocyte lineage, no other structural or numerical aberrations of choroidal or cutaneous melanocytes were yet apparent in F_1_ animals at two months of age. However, sectioning the eyes of 5-month-old transgenics revealed thickening of the choroid by pigmented cells (Supplementary Figure S3C, S3D) in 70% of transgenic animals (n=10). No structural changes were observed in the RPE, apart from pigmentation rescue, which suggests though an activity of the construct in RPE cells too. To confirm that the observed thickening of the choroid was caused by the proliferation of uveal melanocytes, sections were bleached prior to hematoxylin and eosin (H&E) staining. This revealed multiple layers of cells in the choroid, distinguished by their nuclei, and with an overall morphology reminiscent of uveal naevocytes in man (Supplementary Figure S3F), indicating that the diffuse thickening of the choroid is a consequence of a proliferative event.

### Co-operation of oncogenic GNAQ^Q209P^ with p53 loss-of-function induces UM development

**Figure 4:**
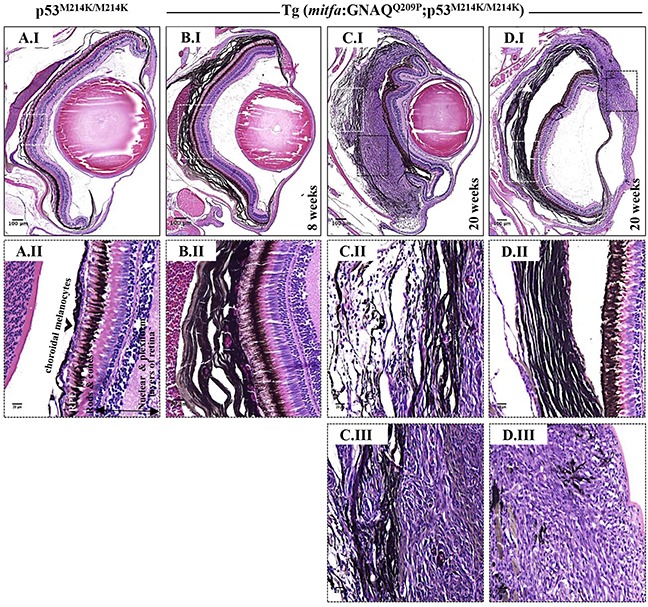
Co-operation of oncogenic GNAQ^Q209P^ and p53 loss-of-function results in UM development Oncogenic GNAQ^Q209P^ Tol2 construct was injected into p53-deficient (p53^M214K/M214K^) zygotes, animals were sacrificed at 2 and 5 months or sooner if ocular protrusion was prominent, then fixed eye specimens were processed and paraffin-embedded for transverse sectioning at a thickness of 5 μm, followed by H&E staining. Representative images are shown. **A.I.** A non-injected p53-deficient (p53^M214K/M214K^) control zebrafish illustrating a single layer of choroidal melanocytes. **B.I.** Benign choroidal hyperplasia in an 8-week-old Tg (*mitfa*:GNAQ^Q209P^;p53^M214K/M214K^) zebrafish. Note the diffuse thickening of the entire choroid caused by the proliferation of choroidal melanocytes. Nuclear and plexiform layers of the retina, photoreceptors layer, and RPE are structurally normal. Also, sclera is respected, with no evidence of infiltration or perforation. **C.I.** An example of malignancy, with non-pigmented hyperproliferative atypical cells developing within an area that shows evidence of choroidal hyperplasia in a 20-week-old Tg (*mitfa*:GNAQ^Q209P^;p53^M214K/M214K^) zebrafish. **D.I.** A second example of a 20-week-old Tg (*mitfa*:GNAQ^Q209P^;p53^M214K/M214K^) zebrafish developing an eye malignancy infiltrating the ciliary body, iris, and cornea, also with evidence of choroidal hyperproliferation. **A.II, B.II, C.II, D.II.** Magnifications of the regions within the white dashed boxes in A.I, B.I, C.I, D.I, respectively, illustrating the changes within the choroidal melanocyte layer (B.II, C.II, D.II), as compared to a structurally normal choroid in a non-injected control (A.II). (**C.III, D.III**) Magnifications of transformed uveal melanocytes depicted in the black dashed boxes in C.I and D.I, respectively. Abbreviations: RPE, retinal pigmented epithelium. Scale bar lengths, as indicated.

**Figure 5:**
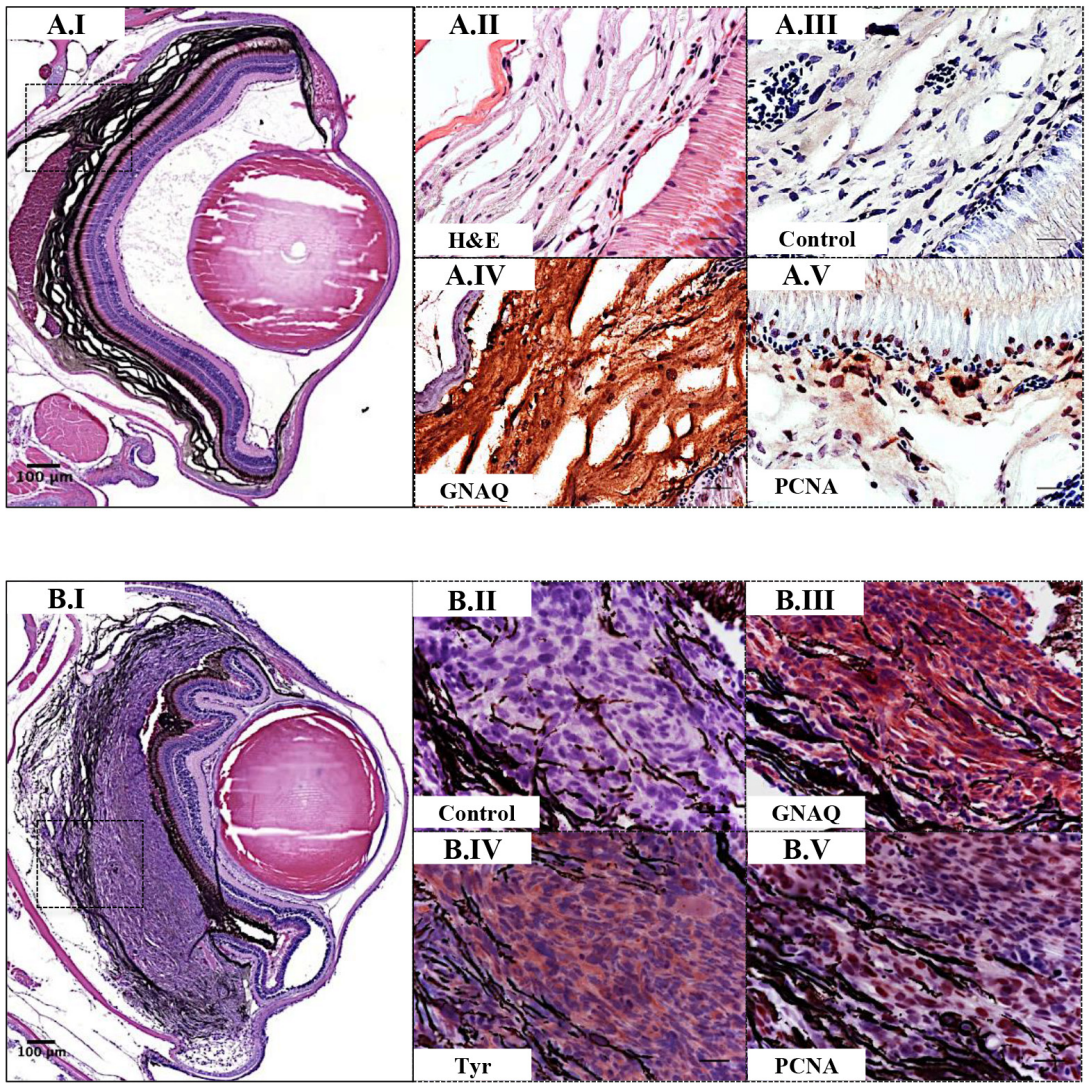
Validation of GNAQ^Q209P^-driven choroidal hyperplasia and uveal tumours in Tg (*mitfa*:GNAQ^Q209P^;p53^M214K/M214K^) zebrafish Transverse sections of formalin-fixed and paraffin-embedded zebrafish eye tissues were stained by IHC, visualized by ImmPact NovaRed peroxidase (HRP) substrate then counterstained with hematoxylin (blue). Representative H&E and IHC images are shown for the pre-malignant **A.I-A.V.** and malignant **B.I-B.V.** stages. (A.I) H&E staining illustrating a 5x magnification of a benign choroidal hyperplasia in a 2-month-old Tg (*mitfa*:GNAQ^Q209P^;p53^M214K/M214K^) zebrafish. (A.II-A.V) Magnifications of the region depicted in the black dashed box in A.I. (A.II) H&E staining of a melanin-bleached section, revealing multiple cellular layers of the thickened choroid. (A.III) Negative control section for IHC staining. (A.IV) GNAQ expression in thickened choroid. (A.V) Hyperplastic choroidal melanocytes are positive for PCNA (dark brown nuclei), especially at the junctional zone between RPE and choroid. (B.I) H&E staining illustrating a 5x magnification of a uveal tumour developing in a 5-month-old Tg (*mitfa*:GNAQ^Q209P^;p53^M214K/M214K^) zebrafish. (B.II) Negative control section for IHC staining. (B.III) Malignant cells expressing GNAQ. (B.IV) Positive immunoreactivity to the melanocytic differentiation marker tyrosinase, indicating the melanocytic origin of malignant cells. (B.V) Numerous PCNA-positive nuclei (dark brown) in malignant melanocytes. Abbreviations: Tyr, tyrosinase; PCNA, proliferating cell nuclear antigen. Scale bars, 100 μm (A.I, B.I); 20 μm (A.II-A.V; B.II-B.V).

Interaction between an oncogenic driver and inactivation of p53 signalling has been mooted for malignant transformation of uveal melanocytes. To investigate this potential, we injected the oncogenic GNAQ^Q209P^ transgene construct into zygotes from homozygous p53^M214K/M214K^ zebrafish mutants whose hypomorphic p53 closely resembles mutant forms isolated from human cancer [30]. As before, only animals with fluorescent lenses as embryos were retained. Again, those animals showed obvious choroidal hyperplasia; however, this was apparent earlier, that is at 2 months (Figure 4B.I). Bleaching of melanin of hyperproliferative choroid revealed multiple cellular layers (Figure 5A.II), and IHC staining confirmed GNAQ overexpression in hyperplastic cells (Figure 5A.IV). However, sectioning a cohort of fifteen F_0_ Tg (*mitfa*:GNAQ^Q209P^;p53^M214K/M214K^) zebrafish at 5 months of age revealed five (33%) with uveal tumours co-existing with choroidal hyperplasia (Figure 4C.I, D.I), four (26%) displaying choroidal hyperplasia only without evidence of uveal malignancy, and two (13%) developing tumours in the leptomeninges surrounding the hindbrain (Supplementary Figure S4), while the remaining had normal phenotypes. Histological examination of the eye malignancies revealed non-pigmented atypical cells developing in conjunction with heavily pigmented hyperproliferative choroidal melanocytes. Infiltration of the ciliary body, iris, and cornea was detected in one tumour specimen (Figure 4D.I). In contrast, sectioning twelve non-injected p53 mutant zebrafish at 5 months of age did not reveal any phenotypic changes in the uvea (Figure 4A.I). However, zebrafish p53 mutants may occasionally develop malignant peripheral nerve sheath tumours (MPNSTs) starting at 8.5 months of age [30], thus it was crucial to verify the origin of the observed eye tumours. To confirm that the malignancies observed following injection of GNAQ^Q209P^ transgene were linked to transgene expression, uveal tumour specimens were examined for GNAQ expression using IHC. In all samples, GNAQ was uniformly expressed by malignant cells (Figure 5B.III). Tyrosinase, the rate-limiting enzyme controlling the synthesis of melanin pigment, has been validated as one of the key immunohistochemical markers for amelanotic melanomas [31]. IHC analysis revealed the expression of tyrosinase by malignant cells (Figure 5B.IV), thus confirming the melanocytic origin of tumours. Furthermore, the transformed melanocytes were uniformly positive for the proliferation marker proliferating cell nuclear antigen PCNA (Figure 5B.V). This contrasted with PCNA being restricted to the interface between the choroid and RPE in pre-malignant lesions expressing GNAQ^Q209P^ (Figure 5A.V). Therefore, in parallel with cutaneous naevi, the junctional zone between the epithelium and supporting connective tissue appears to be a region of active proliferation in uveal naevi. Significantly, early CM develops at this junctional zone [32] and, in keeping, it is likely that this junctional zone also represents the site of origin of UM.

### Nuclear YAP and not ERK phosphorylation correlates with malignant progression of uveal melanocytes

**Figure 6:**
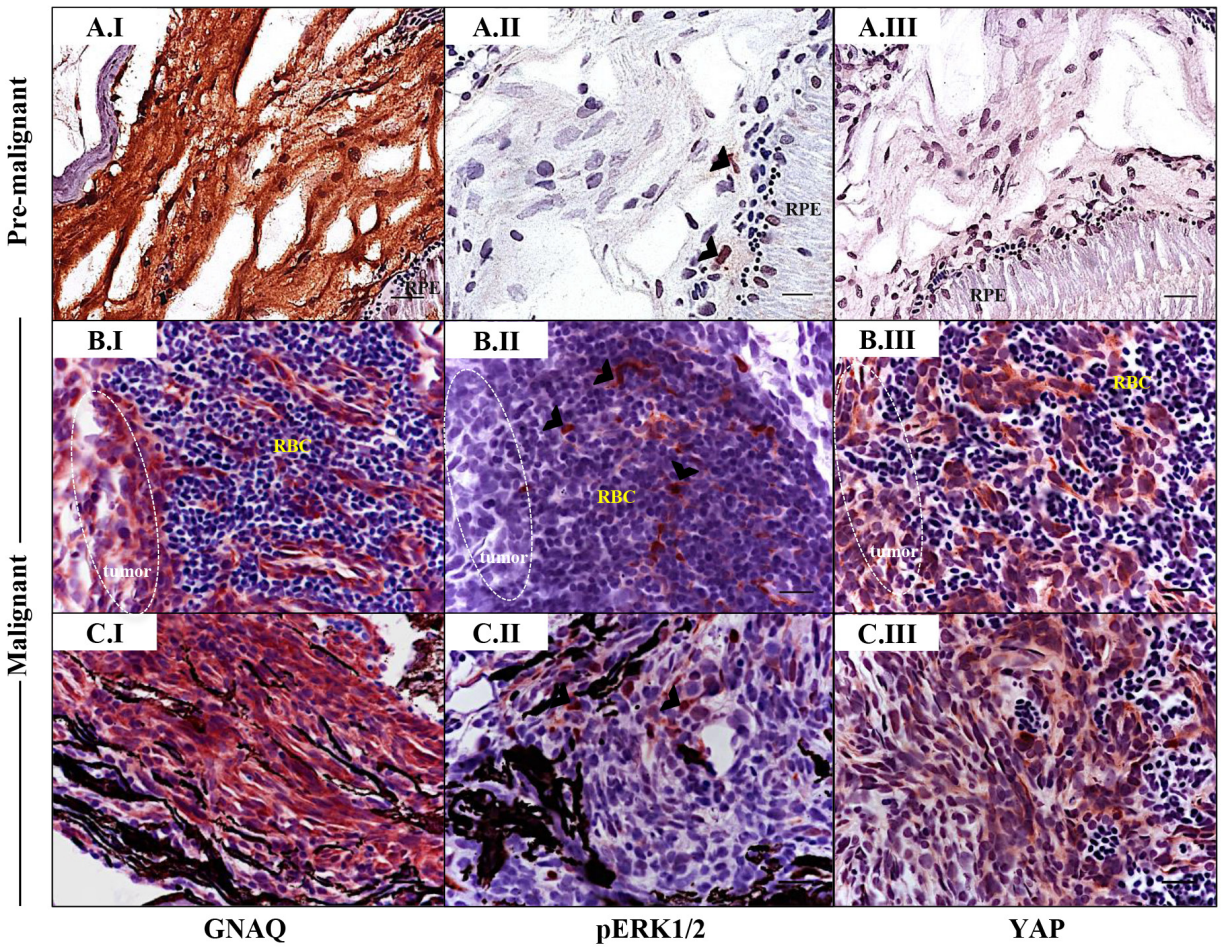
Sporadic ERK activation contrasted with ubiquitous nuclear YAP in malignancies in Tg (*mitfa*:GNAQ^Q209P^;p53^M214K/M214K^) zebrafish Sections of formalin-fixed and paraffin-embedded zebrafish eye specimens were stained for GNAQ, pERK1/2, and YAP by IHC, visualized by ImmPact NovaRed peroxidase (HRP) substrate then counterstained with hematoxylin (purple nuclei). **A.I-A.III.** Representative IHC images of benign hyperproliferative choroid. (A.I) Uniform expression of GNAQ in hyperplastic choroidal melanocytes. (A.II) Only a few cells (black arrowheads) are immunoreactive to pERK1/2. (A.III) Comparatively, more cells displayed nuclear YAP (brown nuclei), although again, this was more noticeable for cells residing at the interface. **B.I-B.III; C.I-C.III.** Representative IHC images of malignant choroidal melanocytes in two independent GNAQ^Q209P^-driven uveal tumours. (B.I, C.I) Transformed melanocytes expressing GNAQ. (B.II, C.II) Malignant cells showing only sporadic immunoreactivity to pERK1/2 (cells showing positive immunoreactivity are indicated with black arrowheads). (B.III, C.III) Ubiquitous YAP nuclear localization (brown nuclei) in transformed uveal melanocytes. Images are representative of sections from three animals. Abbreviations: RPE, retinal pigmented epithelium; RBC, red blood cells. Scale bars, 20 μm.

As revealed by IHC, pERK1/2-positive cells were sporadic in pre-malignant choroidal melanocytes expressing GNAQ^Q209P^, again within the junctional zone (Figure 6A.II), and also in GNAQ^Q209P^-expressing frank uveal tumours (Figure 6B.II, 6C.II). Nuclear YAP was also observed in pre-malignant uveal melanocytes, and again found to be enriched at the junctional zone (Figure 6A.III), but in contrast to pERK1/2, was ubiquitous in malignant uveal melanocytes (Figure 6B.III, 6C.III). Of note, strong immunoreactivity to pERK1/2 was readily detected in cutaneous tumours driven by the expression of oncogenic HRAS^G12V^ (Supplementary Figure S5C.V), indicating its capacity to maintain sustained ERK activation, which was barely evident in comparative GNAQ^Q209P^-driven uveal tumours (Supplementary Figure S5D.V).

**Figure 7:**
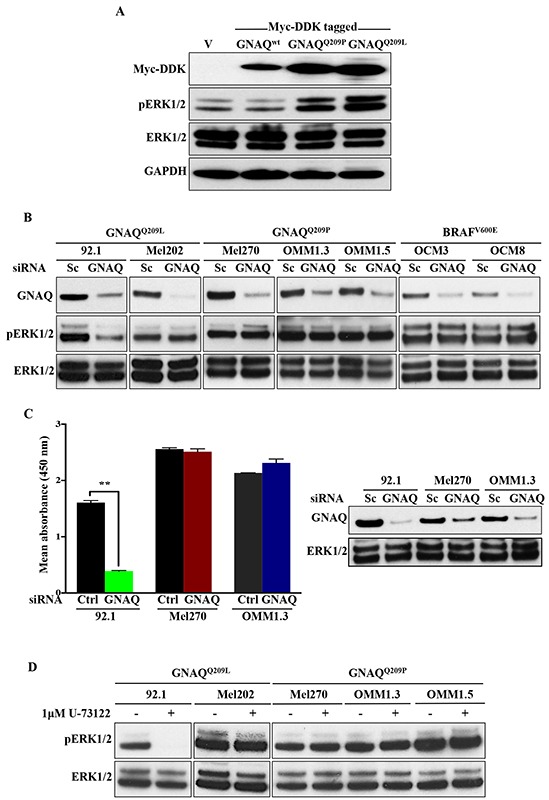
ERK1/2 activation in human UM cell lines harbouring GNAQ mutations is predominantly independent of GNAQ signalling **A.** HEK 293 cells were transfected with vector alone (v) or plasmids encoding Myc-DDK tagged wild-type GNAQ or mutagenized GNAQ^Q209P/L^. 48 hours later, expression of constructs was validated by immunoblotting whole cell lysates with anti-MycDDK antibody. Lysates were also probed for pERK1/2and total ERK1/2, revealing increased pERK1/2 levels upon expression of mutant, but not wild-type GNAQ or empty vector. GAPDH was used as a loading control. **B.** The indicated UM cell lines were transfected with control scrambled (Sc) siRNA or pre-designed and validated siRNA targeting human GNAQ. 48 hours post-transfection, cells were lysed and immunoblotting was performed for endogenous GNAQ, pERK1/2, and total ERK1/2. Knockdown of GNAQ in GNAQ^Q209P^-expressing UM cell lines (Mel270, OMM1.3, and OMM1.5), GNAQ^Q209L^-expressing UM cell line (Mel202), as well as OCM3 and OCM8 lines (expressing BRAF^V600E^; negative controls) was not associated with any alterations in pERK1/2 levels, as compared to cell lines transfected with Sc siRNA. For 92.1 only (expressing GNAQ^Q209L^), pERK1/2 levels were reduced upon GNAQ knockdown. **C.** As detected by pERK1/2 sandwich ELISA, knockdown of GNAQ in 92.1 cell line was associated with a significant decrease in pERK1/2 levels, but didn't affect pERK1/2 levels in Mel270 or OMM1.3. The corresponding immunoblot validates knockdown of GNAQ in the tested UM cell lines, as well as total ERK1/2 levels. Results represent the mean of absorbance readings at 450 nm ± SEM of three technical replicates. Data were analyzed for statistical significance using independent sample t-test at P <0.05. **D.** Treatment of the indicated UM cell lines withU-73122 (1μM), a PLC inhibitor, abolished ERK activation in 92.1 only, whereas the other cell lines showed steady levels of pERK1/2. Abbreviations: Sc, scrambled; Ctrl, control.

To further explore the link between Gαq hyperactivity and activation of ERK signalling, we transiently transfected human embryonic kidney (HEK) 293 cells with empty vector, or vector encoding wild-type GNAQ (GNAQ^WT^), GNAQ^Q209P^, or GNAQ^Q209L^. Only the oncogenic forms (GNAQ^Q209P/L^) resulted in ERK activation (Figure 7A), indicating that transient expression of oncogenic GNAQ can stimulate ERK. To determine whether oncogenic GNAQ signalling is required to maintain ERK activity, we next undertook knockdown of GNAQ in five established human UM cell lines: two expressing oncogenic GNAQ^Q209L^ (92.1 and Mel202), three expressing oncogenic GNAQ^Q209P^ (Mel270, OMM1.3, and OMM1.5), as well as two other cell lines expressing oncogenic BRAF^V600E^ (OCM3 and OCM8). Following GNAQ knockdown, pERK1/2 levels remained unchanged in Mel202, Mel270, OMM1.3, and OMM1.5 cells, and as expected in OCM3 and OCM8, but it was only the 92.1 cell line that responded differently, showing a marked decrease in pERK1/2 levels (Figure 7B). These effects were confirmed in a subset of UM cell lines by the enzyme-linked immunosorbent assay (ELISA) which demonstrated a significant decrease in pERK1/2 levels in GNAQ-silenced 92.1 cell line only, whereas no significant alterations were observed in pERK1/2 levels in GNAQ-silenced Mel270 or OMM1.3 (Figure 7C). Moreover, treatment of UM cell lines with U-73122, a potent and selective PLC inhibitor, was not associated with any changes in pERK1/2 levels, excluding again 92.1 which showed reduced pERK1/2 levels (Figure 7D).

## DISCUSSION

Using the Tol2-based transposon system, we genetically engineered zebrafish to express an oncogenic form of GNAQ (GNAQ^Q209P^) under the control of the *mitfa* promoter to selectively target melanocytes. Despite the transgene being expressed throughout the melanocyte lineage, including in the skin, it was uveal (choroidal) melanocytes that demonstrated hyperproliferation (and presumably leptomeningeal melanocytes), and interestingly, it is precisely this subset of melanocytes in humans that predominantly develops into melanoma in response to GNAQ mutation. Conversely, expression of oncogenic NRAS^Q61L^ or BRAF^V600E^ from the same promoter resulted in hyperproliferation of cutaneous but not uveal melanocytes. Again, in humans, it is epidermal melanocytes that develop into melanoma in response to NRAS or BRAF mutations. Thus, despite being unable to target transgenes specifically to uveal melanocytes, nevertheless, owing to the intrinsic selective responses of anatomical subsets of melanocytes to various oncogenic drivers recurrently mutated in melanoma, we were able to induce an early oncogenic event within the uveal melanocytes, with no interference from the RPE or cutaneous melanocytes.

Upon expression of GNAQ^Q209P^ in the melanocytes of zebrafish *golden* mutants, another effect we observed was its ability to rescue the reduced pigmentation caused by an inactivation of the *slc24a5* gene required for melanosome formation and/or function [29]. This feature was observed in cutaneous melanocytes and RPE. In the mouse, hypermorphic GNAQ/11 alleles also resulted in darker skin and hair by two independent mechanisms: Gαq stimulation increased the proliferation of mouse embryonic dermal (but not epidermal) melanoblasts by bolstering signalling from the endothelin receptor B [33]. Gαq proteins also stimulated melanin synthesis by hair-follicle associated melanocytes, resulting in darker hairs [34]. Hypermorphic Gαq proteins that affect mouse coat colour were still capable of GTP hydrolysis and were not reported to be oncogenic. In contrast, GNAQ^Q209L^ is compromised in GTP hydrolysis and is oncogenic [6, 10, 35]. Its forced expression in mouse melanocytes again resulted in expansion of dermal melanocytes, but to a reduction in melanin transfer to keratinocytes in the hair resulting in their greying [35]. In zebrafish, despite the requirement for endothelin receptor B signalling in pigment pattern formation [36], forced expression of GNAQ^Q209P^ did not appear to affect the proliferation of cutaneous melanocytes. The lack of a conserved proliferative response to stimulation of Gαq signalling between mouse and zebrafish cutaneous melanocytes may reflect a technical difference between the models (for example, timing, duration or amplitude of stimulation achieved), but might also reflect fundamental differences between cutaneous melanocytes in the two species: predominantly dermal in mouse, but also in hair follicles; while predominantly in the hypodermis in zebrafish, but also in the dorsal epidermis (in man, cutaneous melanocytes are predominantly in the epidermis). However, the involvement of Gαq in melanin synthesis appears conserved, although the mechanism is as yet unclear. Potentially Gαq proteins promote the formation or functioning of melanosomes.

Oncogenic GNAQ^Q209P^ alone was not sufficient to drive malignant transformation of uveal melanocytes. However, in co-operation with p53 loss-of-function, GNAQ^Q209P^ was able to promote uveal neoplasia. This is consistent with previous studies reporting the relatively weak oncogenic potential of mutant GNAQ/11 and their ability to transform only immortalized melanocytes that have been genetically altered to be deficient in the p53 and p16/CDK4/RB pathways [6]. Further, the vast majority of uveal melanocytic neoplasms with GNAQ/11 mutations are benign, indicating that they require additional mutations to acquire metastatic potential. In the mouse, however, GNAQ^Q209L^-expressing uveal melanocytes transformed readily and even developed distant metastases without the requirement to engineer further genetic modifications [35]. This could be explained on the basis that spontaneous abrogation of p53 function may be efficient in the mouse model, thus forgoing the requirement to engineer a ‘second hit’, or potentially tumour suppressor mechanisms mediated by p53 pose less significant obstacles to tumourigenesis in mouse uveal melanocytes than in zebrafish and man. A strength of our zebrafish model is therefore the parallels with human UM, as well as the ability to isolate initiation and progression steps in uveal melanocytic neoplasia, which we shall be exploiting in the near future to uncover molecular drivers of UM progression. Of note, a commonality between mice and zebrafish genetically engineered UM models is the occurrence of melanocyte-derived neoplasms developing within the leptomeninges, which is consistent with the previously reported presence of GNAQ/11 mutations in human melanocytomas of the CNS [35, 37, 38].

The UM and CM models we have created in zebrafish demonstrate a distinct dependency on driver mutation that mirrors the pattern of genetic lesions observed in human UM and CM. Oncogenic RAS and BRAF that potently stimulate ERK signalling drive neoplasia of cutaneous melanocytes in zebrafish and are commonly mutated in human CM, but mutations are vanishingly rare in UM. In contrast, oncogenic Gαq drives UM in zebrafish and man and also dermal ‘blue’ naevi in man, but mutations in these proteins are all but absent in CM. Why this pattern exists is not immediately obvious. Certainly, Gαq proteins are expressed in cutaneous melanocytes, and as we and others demonstrate, hyperactivation of Gαq proteins stimulates melanin synthesis in cutaneous melanocytes [34]. Moreover, overexpression of GNAQ^Q209L^ in human cutaneous melanocytes stimulated ERK and contributed to *in vitro* transformation [6], so might be expected to substitute for NRAS or BRAF in CM. What determines the differential sensitivity of uveal and cutaneous melanocytes to the various oncogenic driver mutations may ultimately be resolved by determining the developmental signalling networks operating in these anatomically and ontogenetically distinct melanocyte subsets.

Progression to UM in zebrafish was associated with enhanced YAP rather than ERK signalling. Only a small percentage of uveal melanocytes benign and malignant expressing GNAQ^Q209P^ showed evidence of ERK activation, as revealed by immunoreactivity to pERK1/2. One explanation for this observation is that the effect of GNAQ on ERK1/2-MAPK activation is restricted to a minority of cells, consistent with our findings from in *vitro* experiments, where knockdown of GNAQ or inhibition of PLC-β did not affect pERK1/2 levels in the majority of oncogenic GNAQ-expressing UM cells. What limits the ability of oncogenic GNAQ to activate ERK signalling in the majority of transformed uveal melanocytes is currently unclear. An alternative explanation is that Gαq activates ERK only in short bursts. Certainly, Gαq-mediated activation of PLC-β is transient [39]. In contrast, other targets of Gαq proteins capable of functioning independently of PLC-β, as a consequence of Gαq-mediated activation of Trio and Rho GTPases, such as JUN and YAP, might be activated chronically and substitute for ERK signalling in driving cell proliferation [10, 11, 40].

To conclude, the genetically engineered zebrafish UM model we describe here joins the genetically engineered mouse models [35, 41, 42] as invaluable in *vivo* platforms for establishing the molecular drivers of UM. Particular strengths of our zebrafish system are the parallels with human UM development, technical simplicity, low cost with which genetic modification can be achieved, as well as the ability to segregate benign and malignant stages of neoplastic progression that should facilitate identification of genetic and epigenetic changes propelling this transition. Ultimately, we envisage our model also being used to validate therapeutic targets and establish the efficacy of experimental therapeutic agents.

## MATERIALS AND METHODS

### Zebrafish care and maintenance

Zebrafish were housed at the Biological Services Unit (BSU) at the University of Manchester and maintained at 28.5°C under a 14-hour light/10-hour dark cycle. Embryos were collected and raised in chorion water (60 μg/ml instant ocean salt and 0.01% methylene blue) at 28.5°C up to 5 days post-fertilization (dpf), and thereafter on a recirculating system fed with live and powdered brine shrimps. Transgenic zebrafish expressing oncogenic BRAF^V600E^ [Tg (*mitfa*:BRAF^V600E^)] or HRAS^G12V^[Tg (*mitfa*:HRAS^G12V^)] in the melanocyte lineage have been previously described [18]. Zebrafish husbandry and experimental studies conducted at the University of Manchester were performed in compliance with the Animals (Scientific Procedures) Act 1986 under a Home Office approved project licence.

### Generation of transgenic zebrafish expressing oncogenic GNAQ^Q209P^ and NRAS^Q61L^

#### Recombinant plasmids expressing oncogenic GNAQ^Q209P^ and NRAS^Q61L^

Zebrafish GNAQ cDNA cloned into the gateway-compatible pCMV-SPORT6.1 vector (IMAGp998G1219838Q) was purchased from Source Bioscience. To generate mutant GNAQ^Q209P^, the 872 bp insert was mutagenized using Quickchange II XL site-directed mutagenesis kit (Stratagene). Mutagenic oligonucleotides were designed using NEBase changer software (http://nebasechanger.neb.com), and were as follows: GNAQ^Q209P^_Forward (Fwd): 5′-GATGTCGGGGGTCCACGATCAGAAAG-3′ and GNAQ^Q209P^_Reverse (Rev): 5′-CACCATCCTGAATATGACGCTTTG-3′. GNAQ^Q209P^ middle entry (ME) vector was created by recombining the mutagenized pCMV-SPORT6.1 construct with pDONR221 in a reaction catalysed by BP clonase II plus enzyme mix (Life Technologies) according to the manufacturer's instructions. NRAS^Q61L^ ME vector was created by amplifying NRAS^Q61L^ cDNA from a plasmid kindly supplied by Dr. Piero Crespo (IBBTEC Inicio, Universidad de Cantabria) using primers flanked with *att*B sites, and then recombining with pDONR221 by performing a BP reaction according to the manufacturer's instructions. p5′E-*mitfa* promoter and p3′E-polyA entry clones were kindly provided by Dr. Craig Ceol (UMass Medical School), and Tol2-pDest-*cryaa*:venus-polyA destination vector by Dr. Chi-bin Chien (University of Utah Medical Center). In*vitro* three-fragment recombination reaction between the entry clones (p5′E-*mitfa* promoter, pME-GNAQ^Q209P^ or pME*-*NRAS^Q61L^, and p3′E-polyA) and Tol2-pDest-*cryaa*:venus-polyA destination vector was catalysed using LR clonase II plus enzyme mix (Life Technologies) according to the manufacturer's instructions to generate Tol2-pDest-*cryaa*:venus-polyA;*mitfa*:GNAQ^Q209P^-polyA and Tol2-pDest-*cryaa*:venus-polyA;*mitfa*:NRAS^Q61L^-polyA recombinant expression plasmids. Clones were screened by colony PCR using att sites-specific primers for each of the three fragments, and their sequences were as follows:

p5′E_Fwd: 5′-CTATGACCATGATTACGCCAAGCTA-3′,

p5′E_Rev: 5′-CTGCTTTTTTGTACAAACTTG-3′,

pME_Fwd: 5′-CAAGTTTGACAAAAAAGCAG-3′,

pME_Rev: 5′-CCACTTTGTACAAGAAAGCTG-3′,

p3′E_Fwd: 5′-CAGCTTTCTTGTACAAAGTGG-3′, and

p3′E_Rev: 5′-CAGTGAATTATCAACTATGTA-3′.

Also, correct clones were further verified by sequencing and analyzed using DNA Dynamo sequencing analysis software.

#### Preparation of Tol2 transposase mRNA, microinjection, and screening of injected embryos

Tol2 transposase mRNA was synthesized from a NotI-linearized pCS2-TP plasmid, a kind gift from Dr. Koichi Kawakami (National Institute of Genetics), using the SP6 mMessage mMachine kit (Ambion) then purified using MEGAclear cleanup kit (Ambion). Injection mixture consisted of 5 μl of 0.4 M potassium chloride (KCl), 1 μl of phenol red solution (Sigma-Aldrich), 1 μl of 250 ng/μl Tol2 expression construct, 1 μl of 250 ng/μl Tol2 transposase mRNA, and 2 μl of nuclease-free water (Ambion) to make up a total volume of 10 μl. An approximate volume of one nanoliter was injected into the cytoplasm of one-cell stage fertilized zebrafish embryos. Following injection, embryos were incubated in chorion water at 28.5°C. At 5 dpf, transgenic carriers expressing the Venus green fluorescent protein (GFP) in the eye lens were identified using a Zeiss LumarV12 stereoscope, and images were captured with a Zeiss Axiocam MRc camera. Animals were then raised to adulthood and bred to generate F_1_ generations with stable incorporation of the transgene.

### Fish preparation for histopathological examination

Adult zebrafish were sacrificed in accordance with the schedule 1 guidelines of of the Animal (Scientific Procedures) Act by excess anaesthesia then chemically preserved in 4% paraformaldehyde (PFA) in 1× phosphate buffered saline (PBS) for 2 days at 4°C. Fish were then washed with 1× PBS (3 × 3 minutes) and decalcified in 0.25 M EDTA (pH 8.0) for 2 days at room temperature. Following EDTA treatment, fish were washed with 1× PBS (3 × 3 minutes) then processed using Shandon Excelsior TM carousel-type tissue processor (Thermo Fisher Scientific). Paraffin-infiltrated tissues were carefully embedded in hot paraffin wax using a Tissue-Tek embedding station (Sakura) then allowed to set on a cold plate for 1 hour. Sections were cut at a thickness of 5 μm using RM2035 Microtome (Leica) and subsequently stained with H&E dyes using Thermo Scientific Shandon automatic slide stainer (Thermo Fisher Scientific). Following staining, sections were mounted using Pertex mounting medium (Cell Path) and left to dry overnight at room temperature. Images were acquired with a [*20x/0.80 Plan Apo*] objective using the 3DHistech Pannoramic 250 Flash II slide scanner and processed using 3DHistech Pannoramic viewer software.

### Immunohistochemical staining

Paraffin-embedded tissue sections (5 μm) were initially deparaffinized using xylene then rehydrated through a series of descending concentrations of ethanol. Antigen retrieval was performed by bringing the sections to boil in 10 mM sodium citrate (pH 6.0), and then maintained at a sub-boiling temperature for 20 minutes and room temperature for 30 minutes. Where necessary, melanin was bleached by incubating sections with 3% H_2_O_2_ in 0.05 M phosphate buffer for 30 minutes at 55°C. Otherwise, endogenous peroxidase enzyme was inactivated using 3% H_2_O_2_in distilled water for 10 minutes at room temperature. Sections were then washed with 1x Tris-buffered saline and Tween 20 (TBST) and blocked with 5% normal goat serum/1x TBST for 1 hour at room temperature. Following blocking, sections were incubated with the primary antibody diluted in 5% normal goat serum/TBST according to the manufacturer's instructions and left overnight at 4°C. Primary antibodies used were: rabbit anti-GNAQ (Santa Cruz, 1:100 dilution), rabbit anti-PCNA (Abcam, 1:200 dilution), rabbit anti-tyrosinase (Santa Cruz, 1:100 dilution), rabbit anti-pERK1/2 (Cell Signalling Technology, 1:400 dilution), and rabbit anti-YAP (Cell Signalling Technology, 1:200 dilution). The following day, sections were washed with 1x TBST then incubated with biotinylated goat anti-rabbit secondary antibody (Vector labs) diluted at 1/100 in 1x TBST for 1 hour at room temperature. After washing, sections were incubated with avidin biotin complex (ABC) reagent (Vector labs) for 30 minutes at room temperature then developed with ImmPACT NovaRED Peroxidase (HRP) substrate (Vector labs) for 2 minutes at room temperature, gently washed with tap water, then counterstained with hematoxylin, dehydrated, and finally mounted with Pertex mounting medium (Cell Path) and left to dry overnight at room temperature. Sections were examined using Zeiss Axioplan2 upright microscope. Images were acquired with the AxioCam MRc color CCD camera and processed using AxioVision software.

### Transmission electron microscopy (TEM)

TEM experiments were conducted at the electron microscope (EM) facility of the Faculty of Life Sciences at the University of Manchester. Tissue samples were clipped from the caudal fins of wild-type, *golden*, and transgenic GNAQ^Q209P^ adult zebrafish. Specimens were incubated in a tissue fixative (3% PFA, 3% glutaraldehyde in 0.1 M cacodylate buffer, pH 7.4) for 2 hours at room temperature then overnight at 4°C, and finally post-fixed in 2% osmium tetroxide for 2 hours at 0°C. Specimens were block stained with 0.5% uranyl acetate in 50% ethanol for an overnight then dehydrated in a series of increasing concentrations of ethanol, followed by embedding in Epon 812 resin. Ultra-thin sections (70–80 nm) were prepared using a Reichert-Jung Ultramicrotome. TEM analysis was performed using a FEI Tecnai 12 Biotwin microscope, and photographs were taken using a CCD camera (Eagle, FEI Company). Images were acquired using an Imacon scanner and FlexColor software.

### RNA extraction, cDNA synthesis, and quantitative PCR (qPCR) analysis

Total RNA was extracted from the caudal fin ends of adult zebrafish using TRIzol reagent (Life Technologies), followed by a column-based method of RNA purification using RNeasy mini kit (Qiagen). The purity and concentration of RNA were determined spectrophotometrically by measuring UV absorbances at wavelengths of 260 and 280 nm, using a Thermo Scientific Nanodrop™ 1000. First strand cDNA was then synthesized from 1 μg of total RNA using Tetro cDNA synthesis kit (Bioline). mRNA expression levels were determined using SYBR Green-based real-time quantitative PCR technique. All qPCR reactions were performed on Stratagene MX3000P real-time thermal cycler (Agilent Technologies) using SensiMix SYBR No Rox kit (Bioline). Primers used for qPCR analysis were designed using the PrimerQuest design tool (http://idtdna.com/SciTools). qPCR primer sequences were as follows:

GNAQ_Fwd: 5′-GAACACAATAAGGCCAATGCA-3′,

GNAQ_Rev: 5′-CTGAGAGCTGGTATTCTCGTC-3′,

mitfa_Fwd: 5′-TGTACAGCAATCATGCTCTTCC-3′,

mitfa_Rev: 5′-GTCCCCAGCTCCTTAATTCTGTC-3′,

tyrosinase_Fwd: 5′-CGCAGATGAACAATGGCTC-3′,

tyrosinase_Rev: 5′-AGCAGATACACCCGATGCC-3′,

tryp1_Fwd: 5′-TGCTTGGCGACCCGTCGTTT-3′,

tyrp1_Rev: 5′-GGACGGGCCACGTTACCAGC-3′,

dct_Fwd: 5′-ACCTGTGACCAATGAGGAGATT-3′, and

dct_Rev: 5′-TACAACACCAACACGATCAACA-3′.

Zebrafish β-actin was used as a reference control to normalize gene expression, and had the following sequences: β-actin_Fwd: 5′-TGCGTCTGGATCTAGCT GG-3′ and β-Actin_Rev: 5′-TCCCATCTCCTGCTCGA AG-3′ Reactions were performed in triplicates and the analysis was independently replicated three times. Statistical analysis was performed using two-tailed, unpaired t test (GraphPad Prism software), with the significance level set to P <0.05.

### Cell culture and procedures

HEK 293 cells (a kind gift from Prof. Claudia Wellbrock, University of Manchester) were cultured in L-glutamine/sodium bicarbonate-containing DMEM medium (Sigma-Aldrich). Established human UM cell lines 92.1, Mel270, OMM1.3, and OMM1.5 (kindly gifted by Prof. Sarah Coupland, University of Liverpool) and OCM3 as well as OCM8 (kindly gifted by Dr. Pieter van der Velden, Leiden University Medical Centre) were cultured in L-glutamine/sodium bicarbonate containing-RPMI 1640 medium (Sigma-Aldrich). Both media were supplemented with 10% fetal bovine serum (FBS; Life Technologies) and 1% penicillin & streptomycin (Sigma-Aldrich). All cell lines were maintained at 37°C in a 5% CO_2_ humidified incubator. Cells were grown to 80-90% confluency before passaging. Where indicated, cells were treated with 1 μM U-73122, a PLC inhibitor, for 10 hours.

### Transient transfection

Human Myc-DDK tagged GNAQ cDNA cloned into mammalian pCMV6 vector was purchased from Origene. To generate mutant clones, wild-type GNAQ coding sequence was mutagenized using Quickchange II XL site-directed mutagenesis kit (Stratagene). Mutagenic primers were designed using NEBase changer software (http://nebasechanger.neb.com), and were as follows:

GNAQ^Q209P^_Fwd: 5′-GATGTAGGGGGCCCAAGGTCAGAGAG-3′

GNAQ^Q209L^_Fwd: 5′-GATGTAGGGGGCCTAAGGTCAGAGAG-3′, and

GNAQ^Q209P/L^_Rev: 5′-CTTCTCTCTGACCTTTGGCCCCCTACATCG-3′

One day before transfection, cells were seeded at a density of 2.5 × 10^5^ per well of a 12 well plate format under complete growth conditions. Cells were transfected with plasmid DNA using FuGENE HD (Promega) or small interfering RNAs (siRNAs) using lipofectamine RNAiMAX (Life Technologies) as per the manufacturer's guidelines.

### Western blot analysis

For the preparation of whole protein extracts from cell cultures, cells were washed once with ice-cold 1x PBS then lysed in 1x radioimmunoprecipitation assay (RIPA) buffer (Sigma-Aldrich) freshly supplemented with protease and phosphatase inhibitors (GE healthcare). Samples were agitated for 30 minutes at 4°C then centrifuged at 13,000 rpm for 30 minutes. Following centrifugation, supernatant was collected, and protein content was quantified using bovine serum albumin (BSA) Bradford assay kit (Thermo Fisher Scientific) following the manufacturer's instructions. Protein lysates were mixed with 4x Nupage lithium dodecyl sulfate (LDS) sample buffer (Life Technologies) at a 1:3 (buffer:protein lysate) ratio then denatured by boiling for 5 minutes at 95°C. Whole cell extracts were fractionated by sodium dodecyl sulfate polyacrylamide gel electrophoresis (SDS-PAGE) at 200 volts for 1 hour using XCell SureLock^TM^ Minicell Electrophoresis system (Life Technologies) then transferred onto nitrocellulose membranes (Biorad) using XCell II^TM^ Blot module apparatus (Life Technologies). Membranes were blocked with 5% BSA/1x TBST for 1 hour at room temperature then incubated with an appropriate dilution of the primary antibody for an overnight at 4°C. Primary antibodies used were: rabbit anti-GNAQ (Santa Cruz, 1:1000 dilution), rabbit anti-ERK1/2 (Cell Signalling Technology, 1:2000 dilution), rabbit anti-pERK1/2 (Cell Signalling Technology, 1:2000 dilution), mouse anti-MycDDK (Origene, 1:2000 dilution), and rabbit anti-GAPDH (Cell Signalling Technology, 1:10,000 dilution). To remove excess unbound primary antibody, membranes were washed three times with 1x TBST buffer then incubated with an appropriate horseradish peroxidase (HRP)-conjugated secondary antibody (Cell Signalling Technology, 1:1000 dilution) for 1 hour at room temperature. Finally, signals of specific proteins were detected by a chemiluminescent method using western blotting luminol reagent (Santa Cruz) according to the manufacturer's instructions, and then captured using Optimax film processor. All immunoblotting procedures were performed in triplicate and generated comparable results.

### Detection of endogenous levels of pERK1/2 using enzyme-linked immunosorbent assay (ELISA)

For preparing protein lysates, cells were washed once with ice-cold 1x PBS then lysed in 1x RIPA buffer (Sigma-Aldrich) freshly supplemented with protease and phosphatase inhibitors (GE healthcare). Samples were agitated for 30 minutes at 4°C then centrifuged at 13,000 rpm for 30 minutes. The resulting supernatant (cell lysate) was then collected and analyzed for endogenous levels of ERK1/2 when phosphorylated at Thr202/Tyr204 using PathScan phospho-p44/42 MAPK (Thr202/Tyr204) sandwich ELISA kit (Cell Signalling Technology) following the manufacturer's instructions. Results represent the mean of absorbances at 450 nm ± SEM of three technical replicates. Statistical analysis was performed using two-tailed, unpaired t test (GraphPad Prism software), with the significance level set to P <0.05.

## SUPPLEMENTARY FIGURES


