# Supplementary material for: Minimal contribution of ERK1/2-MAPK signalling towards the maintenance of oncogenic GNAQQ209P-driven uveal melanomas in zebrafish
Source: Oncotarget. 2016 May 6;7(26):39654–70. doi: 10.18632/oncotarget.9207 (PMC5129960; doi:10.18632/oncotarget.9207)
Supplement: Supplementary file 1 [file oncotarget-07-39654-s001.pdf]

SUPPLEMENTARY FIGURES

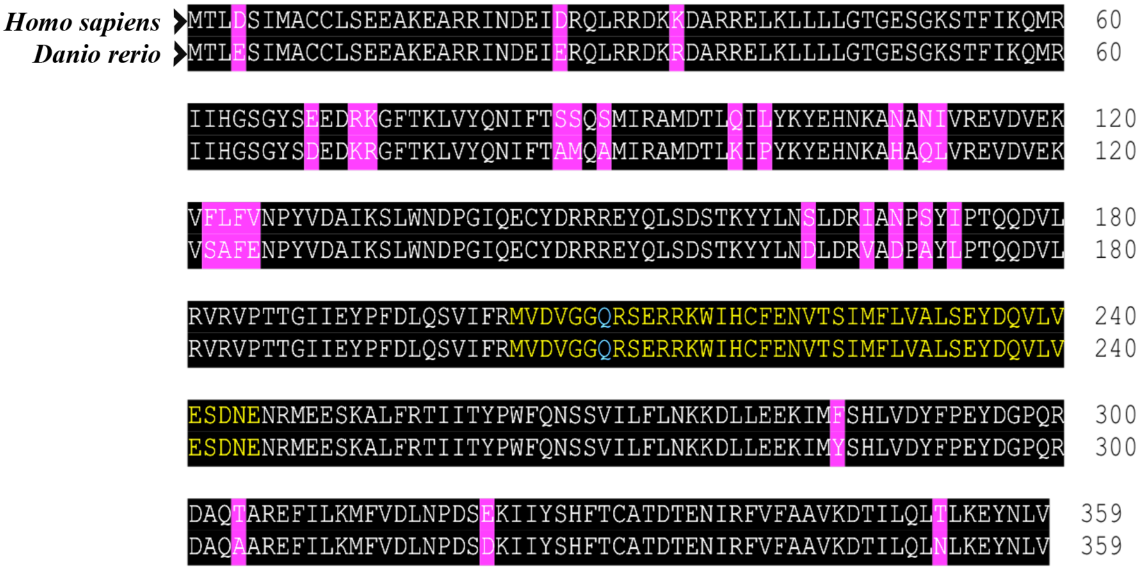

Supplementary Figure S1: Conservation of GNAQ in human and zebrafish genomes. Human (*Homo sapiens*) and zebrafish (*Danio rerio*) GNAQ proteins were identified using NCBI Entrez text search and aligned using BLAST software (http://blast.ncbi.nlm.nih.gov/Blast.cgi) to assess the degree of protein conservation between the two species. Alignment of human GNAQ (NP_002063) and putative zebrafish GNAQ (NP_001138271) amino acid sequences revealed 93% identity (black shading) and a few non-conserved amino acids (pink shading) between the two species. Amino acids encoded by exon 5 are annotated in yellow and are 100% conserved in zebrafish, with Glutamine (Q) at codon 209 annotated in blue.

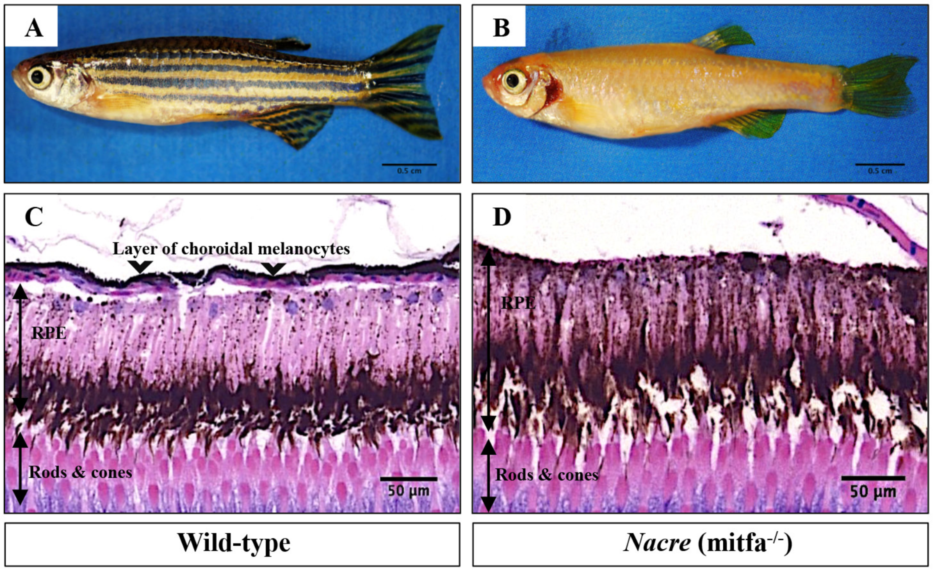

Supplementary Figure S2: *mitfa* is required for the development of zebrafish choroidal melanocytes. (Top panel) Lateral views of adult wild-type **A.** and homozygous *mitfa*$^{-/-}$ (*nacre*; **B.** zebrafish. (Bottom panel) H&E staining of transverse sections of formalin-fixed and paraffin-embedded eye specimens of adult wild-type **C.** and *nacre* **D.** zebrafish. Wild-type tissue section shows a single layer of choroidal melanocytes (black arrowheads). In contrast, choroidal melanocytes are absent in *nacre* mutants. Abbreviations: RPE, retinal pigmented epithelium. Scale bars, as indicated.

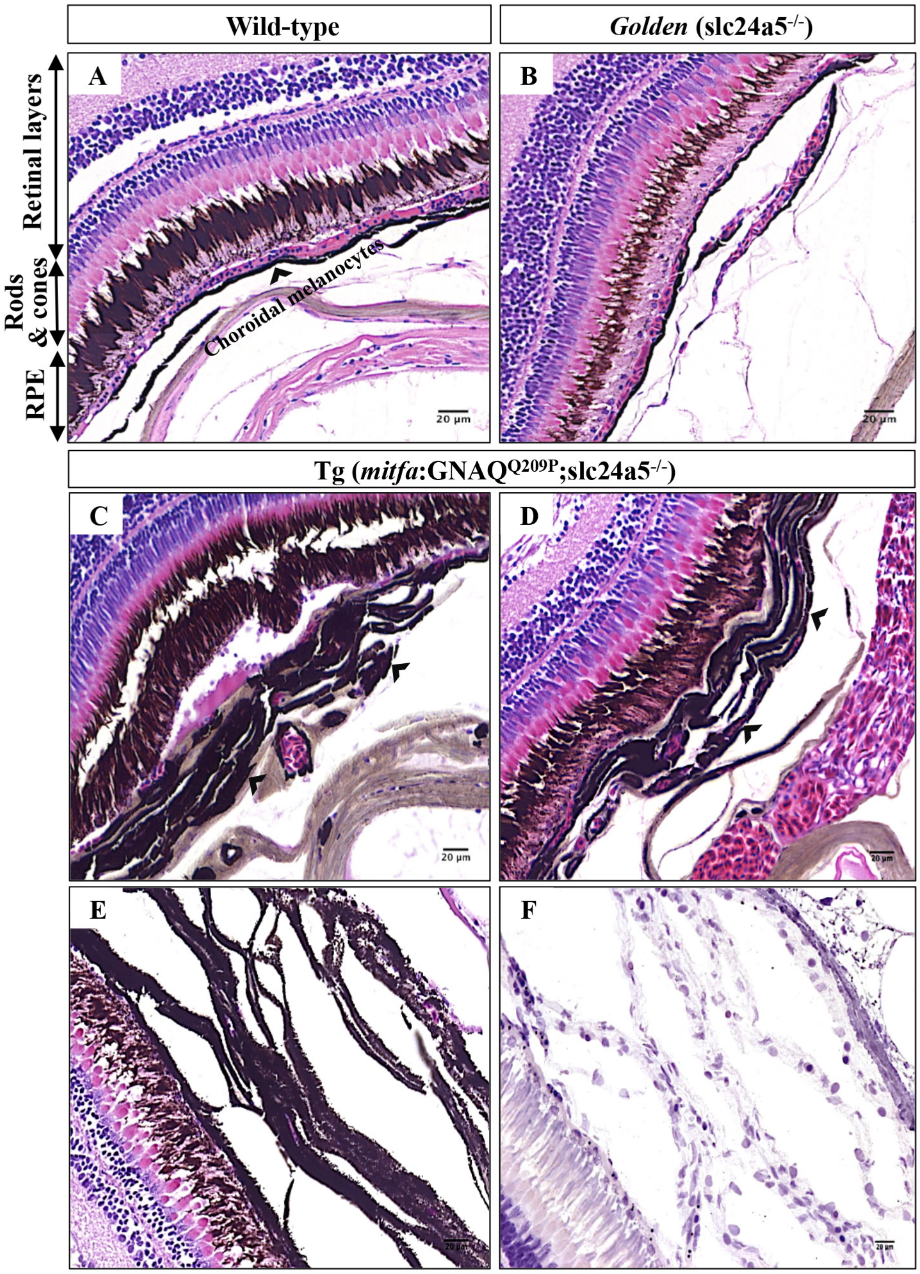

Supplementary Figure S3: Choroidal hyperplasia is evident in pigmentation-rescued Tg (*mitfa*:GNAQ^{Q209P};slc24a5$^{-/-}$) zebrafish. A-D. H&E staining of transverse sections of formalin-fixed and paraffin-embedded eye tissues of a 5-month-old adult zebrafish. (A) Wild-type demonstrating different structures of the zebrafish retina as indicated. (B) *Golden* mutant: note the hypopigmented RPE and structurally normal choroid. (C, D) Tg (*mitfa*:GNAQ^{Q209P};slc24a5$^{-/-}$): note here benign hyperplastic choroidal lesions (black arrowheads) developing beneath pigmentation-rescued RPE. **E.** A representative example of H&E stained transverse section illustrating thickened and heavily pigmented choroidal melanocyte layer before melanin bleaching. **F.** Multiple cellular layers of thickened choroid, as revealed by multiple nuclei (blue; counterstained with hematoxylin) after melanin bleaching. Abbreviations: RPE, retinal pigmented epithelium. Scale bar lengths, as indicated.

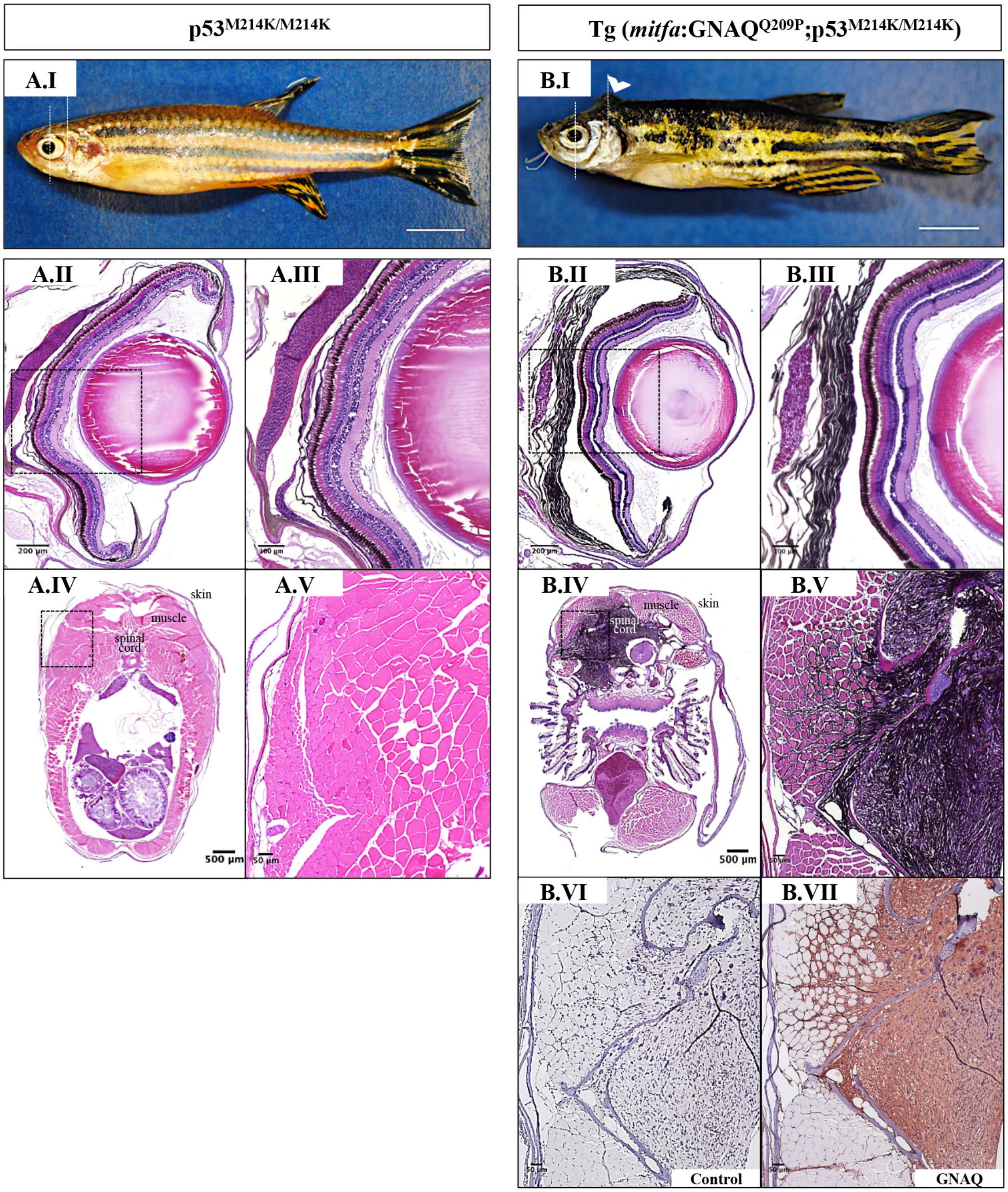

Supplementary Figure S4: Oncogenic GNAQ^{Q209P} expression induces malignant CNS melanoma in p53-deficient zebrafish. A.I, B.I. Lateral views of 5-month-old zebrafish. (A.I) p53-deficient zebrafish (p53$^{M214K/M214K}$). (B.I) Tg (*mitfa*:GNAQ^{Q209P};p53$^{M214K/M214K}$) zebrafish developing masses protruding from the back of the head (white arrowhead). Scale bars, 0.5 cm. Representative images of H&E stained tissue sections through the torso of p53$^{M214K/M214K}$ animals **A.IV, A.V.** and age-matched Tg (*mitfa*:GNAQ^{Q209P}; p53$^{M214K/M214K}$) **B.IV, B.V.** show highly infiltrative, pigmented malignancy appearing to emanate from the leptomeninges surrounding the hind brain of the latter. The same animals were sectioned through the eyes revealing normal development of the choroid in p53$^{M214K/M214K}$ mutants **A.II, A.III.**, but benign hyperproliferation of choroidal melanocytes in Tg (*mitfa*:GNAQ^{Q209P};p53$^{M214K/M214K}$) **B.II, B.III.**. (A.III, A.V, B.III, B.V) Magnifications of the regions depicted in black dashed boxes in A.II, A.IV, B.II, B.IV, respectively. **B.VI.** Negative control section incubated with 1x PBS instead of primary antibody. **B.VII.** Area of pigmented melanocytic infiltration showing GNAQ expression upon IHC staining. Scale bar lengths, as indicated.

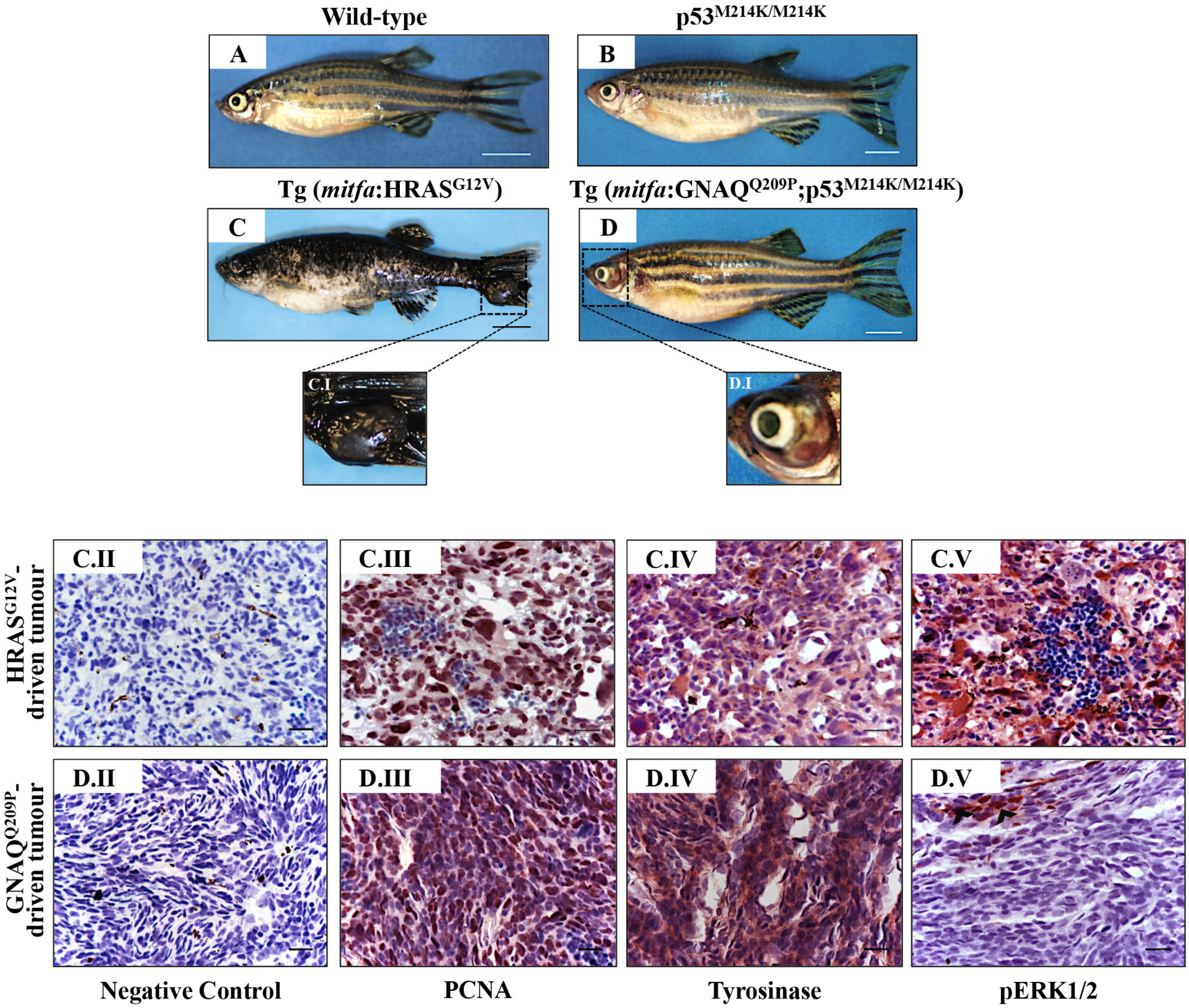

Supplementary Figure S5: Oncogenic GNAQ^{Q209P} and HRAS^{G12V} show different potentials in sustaining ERK1/2-MAPK signalling in melanomas of uveal and cutaneous origins. Lateral views of wild-type **A.** p53$^{M214K/M214K}$ **B.** Tg (*mitfa*:HRAS^{G12V}) **C.** and Tg (*mitfa*:GNAQ^{Q209P};p53$^{M214K/M214K}$) **D.** adult zebrafish. Scale bars, 0.5 cm. **C.I, D.I.** Magnifications of the regions depicted in the black dashed boxes in C and D, respectively. **C.II-C.V.** Representative IHC images of HRAS^{G12V}-driven cutaneous tumours. (C.II) Negative control. (C.III) Numerous melanocytic tumour cells expressing the proliferation marker PCNA: nuclei are stained blue (hematoxylin) and PCNA-positive cells acquire dark brown nuclei. (C.IV) Malignant cells expressing the melanocytic differentiation marker tyrosinase. (C.V) Strong immunoreactivity of transformed cutaneous melanocytes to pERK1/2, indicating the activation of ERK1/2-MAPK signalling. **D.II-D.V.** Representative IHC images of GNAQ^{Q209P}-driven uveal tumours. (D.II) Negative control. (D.III) Malignant cells expressing PCNA (dark brown nuclei).(D.IV) Positive immunoreactivity of transformed cells to tyrosinase, indicating the melanocytic origin of the developed ocular neoplasia. (D.V) Transformed uveal melanocytes are barely immunoreactive to pERK1/2, indicating low activity of ERK1/2-MAPK signalling. Abbreviations: PCNA, proliferating cell nuclear antigen. Scale bars, 20 µm.
